# Supplementary figures and images for: Topological Structures in the Space of Treatment-Naïve Patients with Chronic Lymphocytic Leukemia
Source: Cancers (Basel). 2024 Jul 26;16(15):2662. doi: 10.3390/cancers16152662 (PMC11311631; doi:10.3390/cancers16152662)

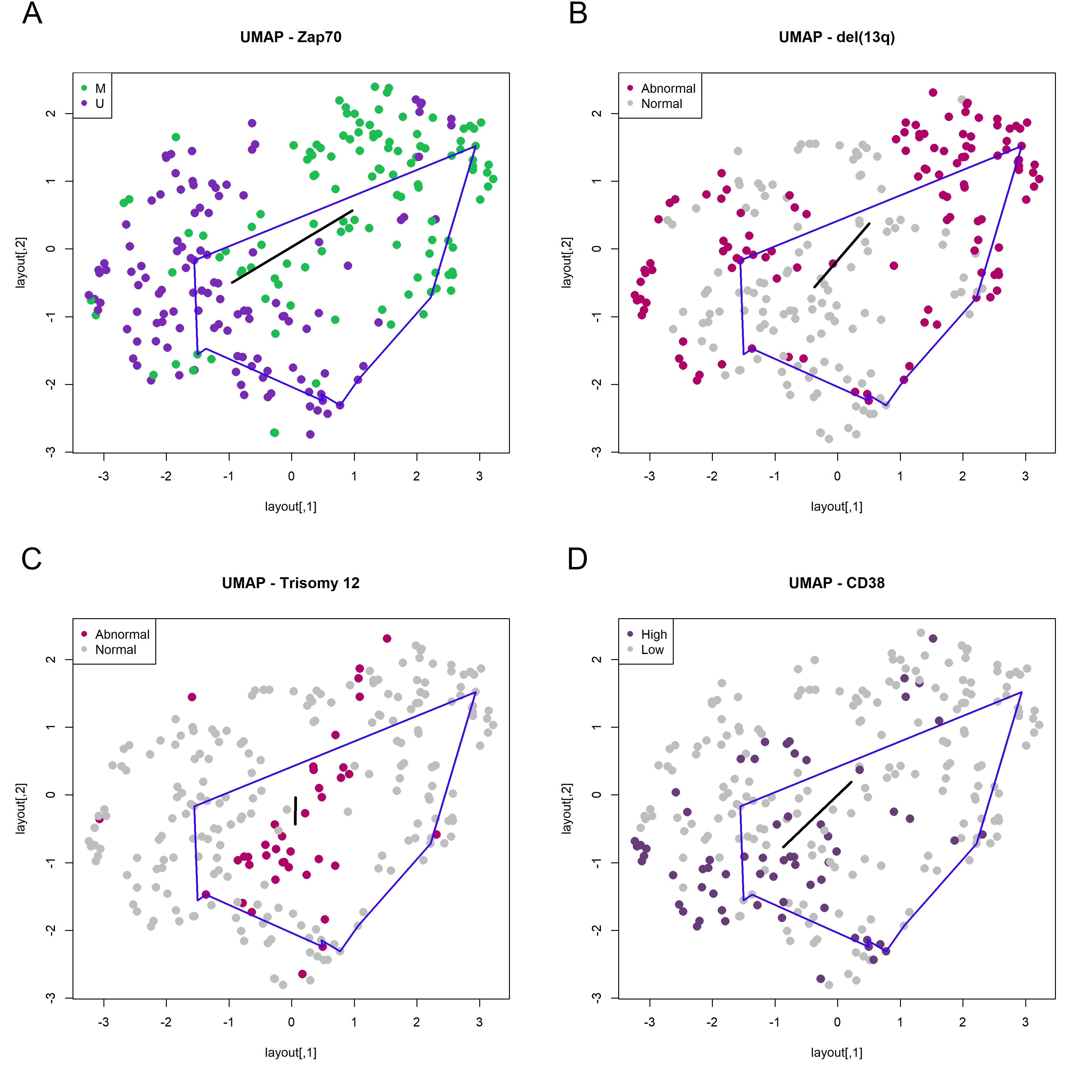

Supplement: Supplementary file 1 [file cancers-16-02662-s001.zip › Figure S1.jpg]

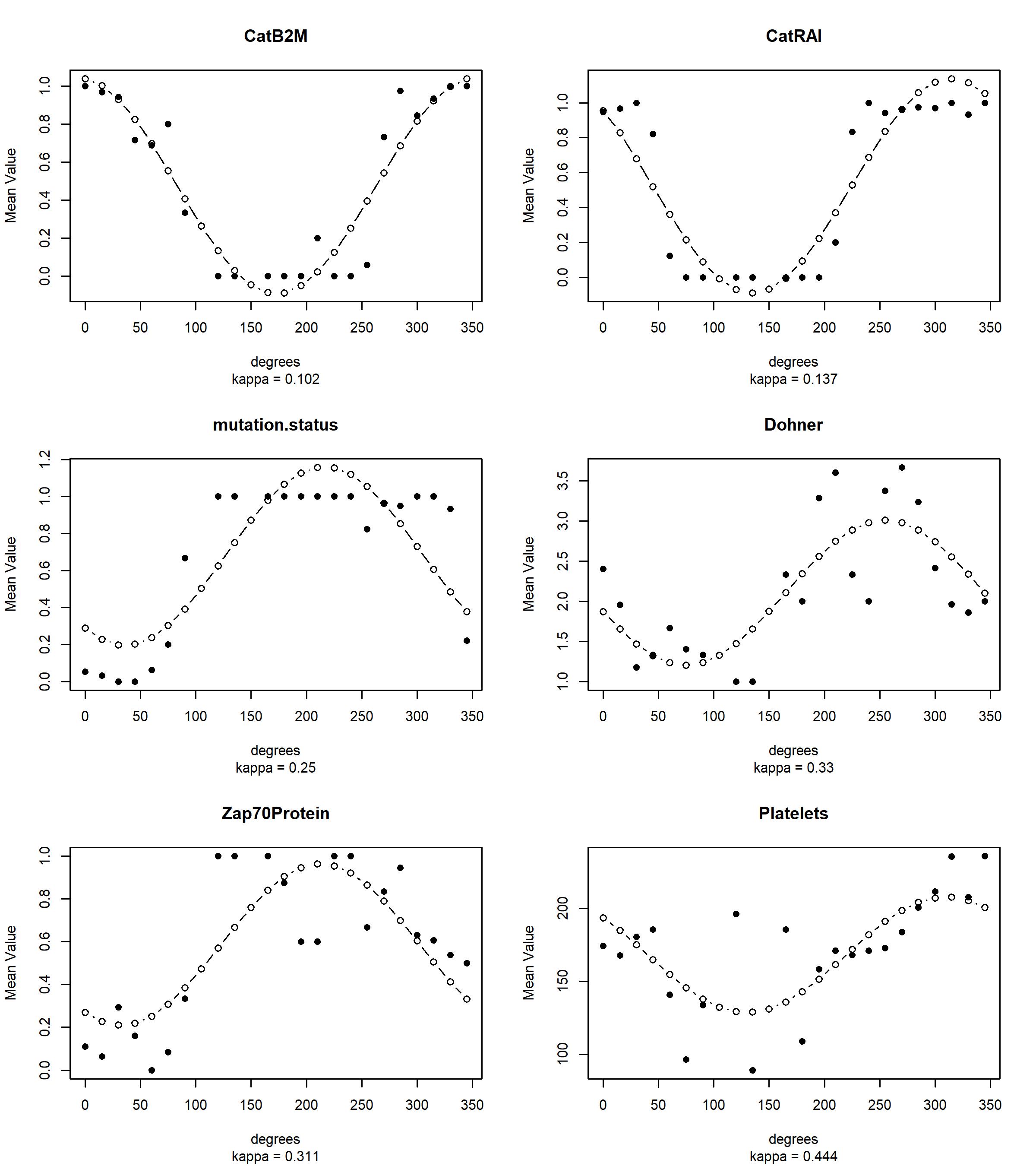

Supplement: Supplementary file 1 [file cancers-16-02662-s001.zip › Figure S2.jpg]

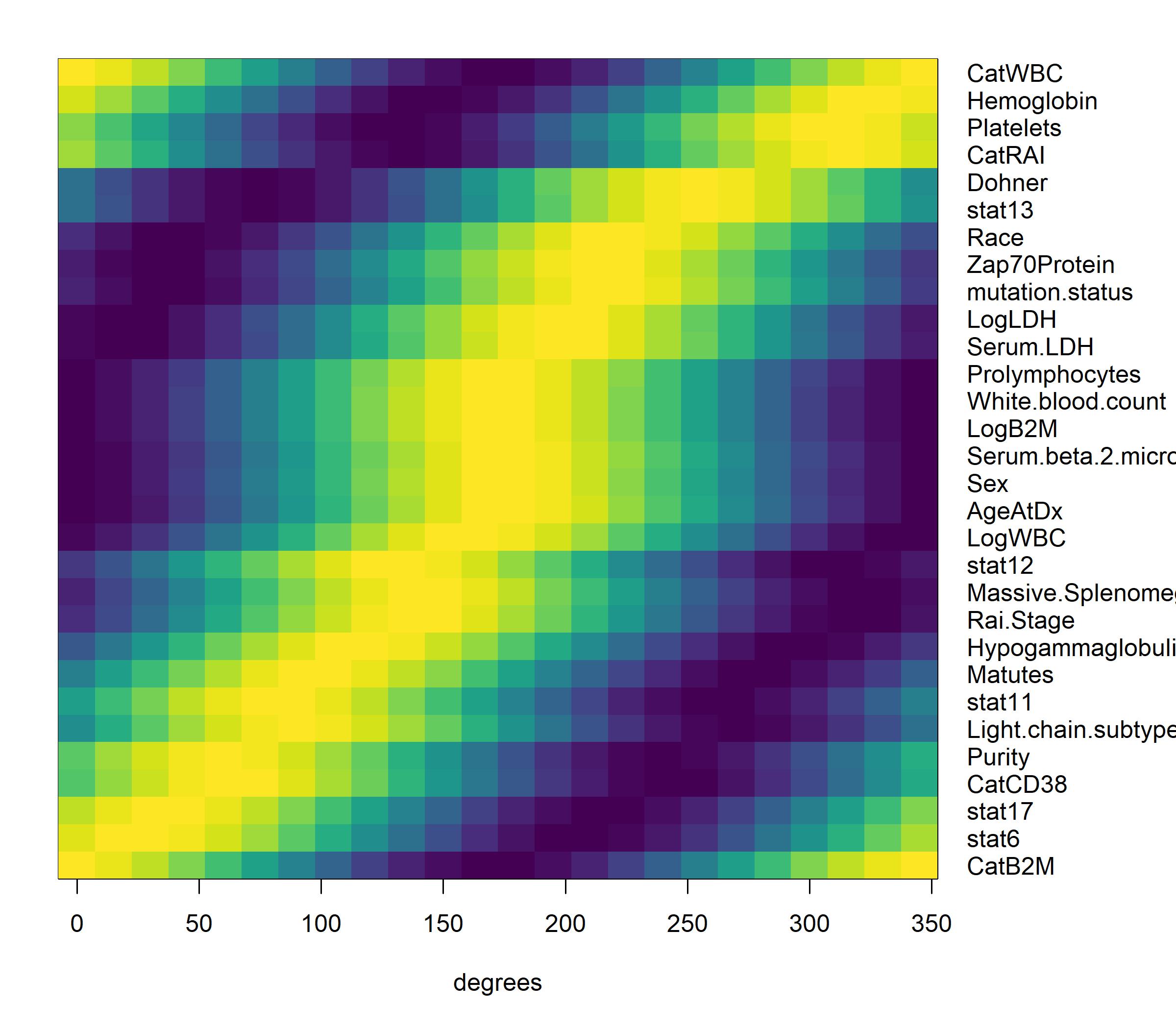

Supplement: Supplementary file 1 [file cancers-16-02662-s001.zip › Figure S3.jpg]

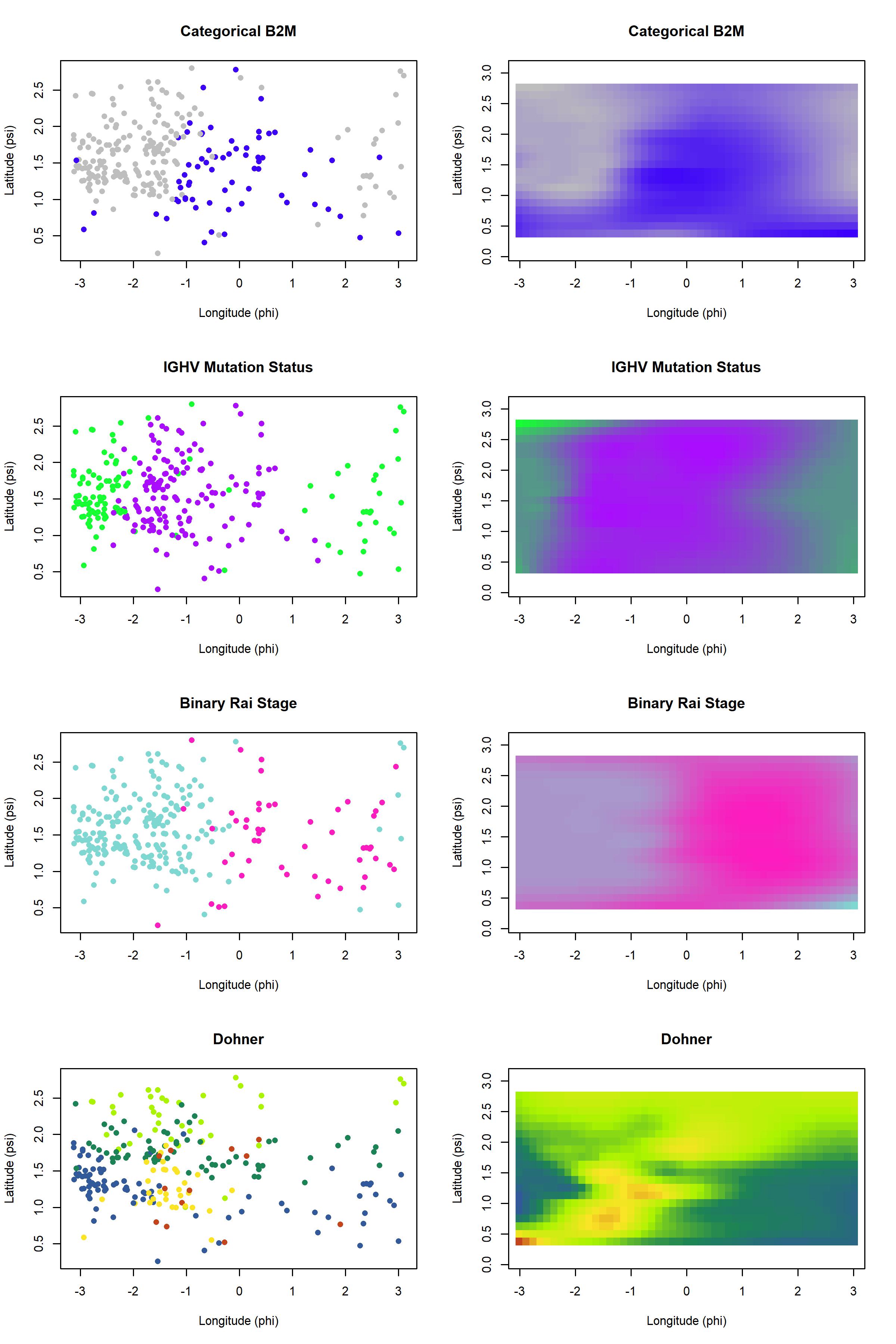

Supplement: Supplementary file 1 [file cancers-16-02662-s001.zip › Figure S4.jpg]

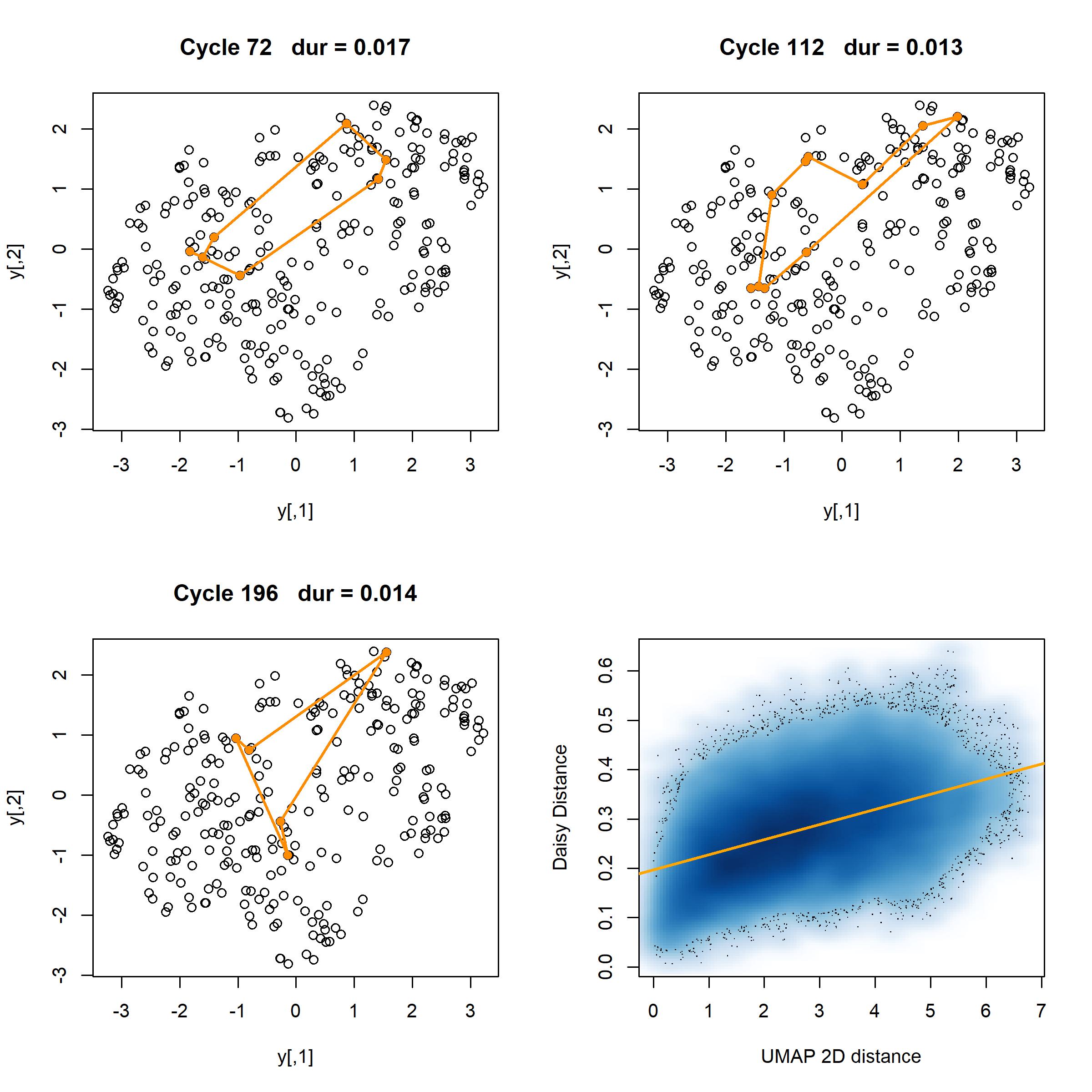

Supplement: Supplementary file 1 [file cancers-16-02662-s001.zip › Figure S5.jpg]
